# Sensitivity Detection of Uric Acid and Creatinine in Human Urine Based on Nanoporous Gold

**DOI:** 10.3390/bios12080588

**Published:** 2022-08-01

**Authors:** Keshuai Shang, Shuangjue Wang, Siyu Chen, Xia Wang

**Affiliations:** 1 State Key Laboratory of Microbial Technology, Shandong University, Qingdao 266237, China; asakas13468242423@163.com (K.S.); wsj782872917@163.com (S.W.); 2 The Faculty of Engineering, Architecture and Information Technology, The University of Queensland, Brisbane, QLD 4072, Australia; siyu.chen2@uqconnect.edu.au

**Keywords:** nanoporous gold, co-catalytic strategy, uric acid, creatinine, human urine

## Abstract

Given the significance of uric acid and creatinine in clinical diagnostic, disease prevention and treatment, a multifunctional electrochemical sensor was proposed for sensitive detection of uric acid and creatinine. The sensitive detection of uric acid was realized based on the unique electrochemical oxidation of nanoporous gold (NPG) towards uric acid, showing good linearity from 10 μM to 750 μM with a satisfactory sensitivity of 222.91 μA mM^−1^ cm^−2^ and a limit of detection (LOD) of 0.06 μM. Based on the Jaffé reaction between creatinine and picric acid, the sensitive detection of creatinine was indirectly achieved in a range from 10 to 2000 μM by determining the consumption of picric acid in the Jaffé reaction with a detection sensitivity of 195.05 μA mM^−1^ cm^−2^ and a LOD of 10 μM. For human urine detection using the proposed electrochemical sensor, the uric acid detection results were comparable to that of high-performance liquid chromatography (HPLC), with a deviation rate of less than 10.28% and the recoveries of uric acid spiked in urine samples were 89~118%. Compared with HPLC results, the deviation rate of creatinine detection in urine samples was less than 4.17% and the recoveries of creatinine spiked in urine samples ranged from 92.50% to 117.40%. The multifunctional electrochemical sensor exhibited many advantages in practical applications, including short detection time, high stability, simple operation, strong anti-interference ability, cost-effectiveness, and easy fabrication, which provided a promising alternative for urine analysis in clinical diagnosis.

## 1. Introduction

Nonprotein nitrogen (NPN) in the human body refers to nitrogenous substances other than protein, mainly including uric acid (UA), creatinine (Cn), creatine (Cr), amino acids, etc. [1]. Among them, uric acid and creatinine are essential metabolites of protein and nucleic acid. Typically, endogenous and exogenous uric acid and creatinine, which are formed and distributed in the body, will be excreted in urine through glomerular filtration. Uric acid is the end-product of purine metabolism in the body and exists in the form of highly soluble urates [2]. The average levels of uric acid in serum and urine of healthy individuals ranged from 240 to 520 μM and 1.44 to 4.43 mM, respectively [3]. An increase or decrease in uric acid levels can trigger or reveal a range of diseases. With the improvement of living standards, people’s diets have changed significantly, and the intake of high purine foods has led to a significant increase in the incidence of hyperuricemia and gout. Related studies have found that high uric acid levels are also closely associated with fatty liver [4], essential hypertension [5], coronary heart disease [6], Parkinson’s disease [7], atherosclerosis [8], metabolic syndrome [9], and many other diseases.

Creatinine, a metabolite of creatine and creatine phosphate, is produced primarily in muscle and released into the bloodstream at a highly constant rate that is proportional to the individual’s muscle mass [10,11,12]. Creatinine is excreted from the kidneys by glomerular filtration and accumulates in the urine without reabsorption through the renal tubules. Normal levels of creatinine range from 45 to 140 μM in serum and 0.8 to 2.0 g/24 h in urine [13]. A sera creatinine value above 500 μM indicates that the patient has severe kidney disease, which can eventually lead to dialysis or transplantation [14], while values below 40 μM indicate a decrease in human muscle mass [15]. Therefore, a serum creatinine level above 150 μM can be used as a marker of renal insufficiency, while below 40 μM can be used as an indicator of muscle dysfunction [16]. On the other hand, urine creatinine can effectively assess the concentration of harmful substances in urine and is often used clinically together with urine microalbumin to assess the degree of kidney damage. Urine creatinine also plays an essential role in assessing the validity of urine samples and dilution [17]. The low creatinine concentrations may indicate attempts to tamper with samples by dilution in drug abuse screening tests. The creatinine level in humans is a direct reflection of renal, muscle, and thyroid function [18], which is of great help in monitoring and diagnosing the condition of patients.

In clinical practice, renal function is usually assessed by monitoring uric acid excretion and creatinine clearance. The use of urinary uric acid to creatinine ratio has led to rapid monitoring of primary gout, renal disease status, hyperuricemia, renal failure, and acute uric acid nephropathy [19], which implies the importance of accurate detection and analysis of uric acid and creatinine levels in human blood and urine. Therefore, it is worthwhile to establish a simple and effective assay for the sensitive and accurate determination of uric acid and creatinine in biological samples for disease diagnosis and health assessment.

The traditional methods for the detection of uric acid and creatinine include spectroscopy [20,21,22], high performance liquid chromatography [23,24], capillary electrophoresis [25,26], and chromatography-mass spectrometry [27,28]. Although these methods have high accuracy and sensitivity, they still suffer from the requirements of expensive instruments and complex operation protocols. In the past decades, electrochemical techniques have been considered as reliable and promising assays because of their specificity, high sensitivity, rapidity, ease of miniaturization, simplicity of operation, and low price [19].

The development and application of modified electrodes with various materials have made the field of electrochemical sensor research flourish [29,30]. Many electrochemical sensors have been developed for the determination of uric acid and creatinine [31,32,33,34]. Among these electrochemical biosensors, non-enzymatic sensors have gained much attention due to their low-cost, simplicity, accuracy and portability [35]. Non-enzymatic electrochemical sensors usually use electrically modified materials as recognition elements. With the development of various novel materials, the performance of non-enzymatic electrochemical sensors has been constantly improved. Various nanomaterials (metal nanoparticles, nanoporous metals, carbon-based materials, polymer films, etc.) are gradually used to fabricate non-enzymatic electrochemical sensors, especially nanoporous metals [35]. Nanoporous metals possess a high specific surface area with ideal pore sizes and size distributions, and high gross pore volume [36,37,38]. It is supposed that the excellent three dimensional nanoporous structure of nanoporous metals may enhance electron transfer and mass transfer of the modified electrodes, which, in turn, may improve the analytical performance of non-enzymatic electrochemical sensors.

Among nanoporous metals, nanoporous gold (NPG) is a widely used nanomaterial for the construction of electrochemical sensors. The three-dimensional nanoporous structure of NPG gives it many excellent properties, including good electrical conductivity, chemical stability, and enhanced response signal [39,40,41,42]. Especially, NPG exhibits excellent electrocatalytic activity properties for many compounds. Owing to the unique electrocatalytic properties of NPG, it was selected as a glass carbon electrode (GCE) modification material to construct NPG/GCE electrode in this study. The detection of uric acid was realized based on the electrochemical oxidation of nanoporous gold (NPG) towards uric acid. Based on the Jaffé reaction between creatinine and picric acid, the detection of creatinine was indirectly achieved by determining the consumption of picric acid in the Jaffé reaction.

## 2. Materials and Methods

### 2.1. Chemicals and Materials

Uric acid (≥99.0%) and creatinine (≥99.0%) were purchased from Dingguo changsheng Biotechnology Co., Ltd. (Qingdao, China). All other chemicals were of analytically pure grade. Deionized water was prepared by Millipore’s Direct-Q 3 UV (Darmstadt, Germany) and used in all experiments. GCEs, saturated calomel electrodes (SCE), and α-Al_2_O_3_ powder (1.0 μm, 0.3 μm, 0.05 μm) were purchased from Shanghai Chenhua Instrument Co., Ltd. (Shanghai, China). All reagents used were of analytical grade.

### 2.2. Preparation of Electrodes

First, GCE was polished sequentially with α-Al_2_O_3_ slurry of different particle sizes (1.0 μm, 0.3 μm, 0.05 μm) and washed by ultrasonic cleaning with ultrapure water and anhydrous ethanol for 1 min, respectively. Then, the GCE was activated by cyclic voltammetry (CV) in 0.5 mol/L H_2_SO_4_ solution. The voltage was scanned from −1.0 V to +1.0 V at a rate of 100 mV s^−1^. Scans were repeated until a stable cyclic voltammetry curve was observed.

NPG films were prepared by dealloying Au/Ag sheets (Au50Ag50, wt%) in concentrated nitric acid at 40 °C for 1 h [43]. After the preparation of NPG, NPG film was coated on the surface of GCE to construct an NPG/GCE electrode. An appropriate amount (4 μL) of Nafion solution (0.5%, wt%) was dripped on the surface of the NPG/GCE electrode to improve the stability of the constructed electrochemical sensor.

### 2.3. Analytical Procedure

The morphology of NPG film was characterized by scanning electron microscopy (SEM, FEI Quanta250 FEG, Corporate Contacts, ThermoFisher SCIENCETIC, Waltham, MA, USA).

Electrochemical measurements were carried out using a CHI760E electrochemical workstation (Shanghai Chenhua Instrument Co., Ltd., Shanghai, China) according to a previous study [44]. All the three-electrode electrochemical measurements involved in this study used the NPG/GCE electrode as the working electrode, the Pt electrode as the counter electrode, and the SCE as the reference electrode.

High-performance liquid chromatography (HPLC) analysis was carried out using HPLC (Shimadzu LC-20A, Tokyo, Japan). The mobile phase consisted of mobile phase A (20 mM ammonium acetate solution) and mobile phase B (methanol) with a ratio of 95:5. The HPLC measurements were carried out with a flow rate of 0.9 mL min^−1^, a column temperature of 35 °C and a detection wavelength of 240 nm.

For human urine analysis, three human urine samples were collected and diluted by ammonium acetate (20 mM) solutions with a ratio of 1:19 for HPLC detection and by phosphate buffer solution (PBS, 50 mM, pH 6.0) with a ratio of 1:9 for electrochemical measurements by the NPG/GCE electrode.

## 3. Results and Discussion

### 3.1. Construction and Characterization of the NPG/GCE

**Figure 1:**
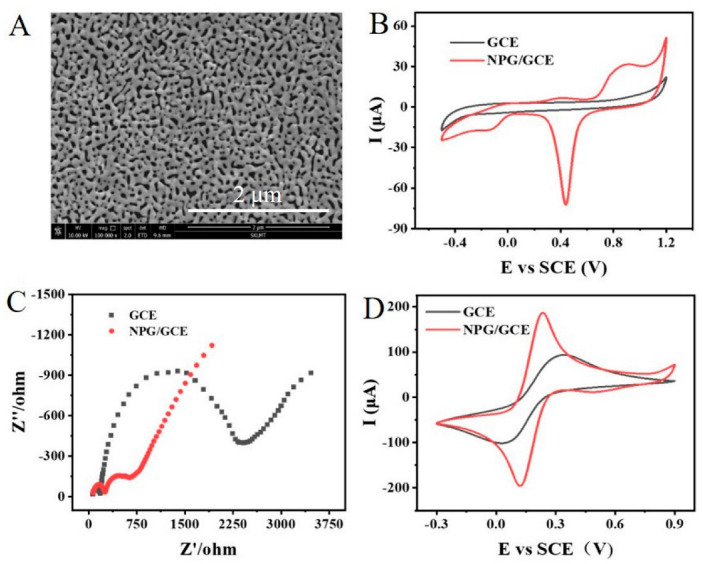
(**A**) The SEM image of NPG at a magnification of 100,000×. (**B**) The CV curves of bare GCE and NPG/GCE in PBS (50 mM, pH 7.0). (**C**) The EIS profiles of bare GCE and NPG/GCE in 5.0 mM K_3_Fe(CN)_6_ solution (frequency range of 0.01 to 106 Hz). (**D**) The CV curves of bare GCE and NPG/GCE in 5.0 mM K_3_Fe(CN)_6_ solution.

NPG was prepared using the dealloying method according to a previous report [43]. The morphology of NPG film was characterized by SEM as shown in Figure 1A. The prepared NPG has nanopores and a continuous gold ligament structure (Figure 1A), which endows NPG with a high specific surface area, excellent electrical conductivity, high electrochemical signal response, and electrocatalytic activity [42]. After NPG preparation, an NPG/GCE electrode was constructed according to the method described in “Section 2.2”.

The prepared NPG/GCE electrode was electrochemically characterized in a three-electrode reaction cell. Figure 1B depicted the cyclic voltammetry (CV) responses of bare GCE and the NPG/GCE measured in a PBS (50 mM, pH 7.0) at a scan rate of 50 mV s^−1^ from −0.6 V to +1.2 V. No redox peaks were observed for bare GCE. In contrast, the NPG/GCE exhibited well-defined redox peaks of NPG with the oxidation peak potential (Epa) and reduction peak potential (Epc) at +0.85 V and +0.45 V, respectively, which indicated that NPG was successfully modified on GCE and showed good electrochemical activity.

The bare GCE and NPG/GCE electrodes were further characterized by electrochemical impedance spectroscopy (EIS) and cyclic voltammetry (CV) in a 5 × 10^−3^ mol/L K_3_Fe(CN)_6_ solution. Comparing the impedance spectra as shown in Figure 1C, the impedance of the NPG/GCE was significantly reduced. In Figure 1D, the CV curves of both GCE and NPG/GCE electrodes showed a pair of obvious redox peaks, including the oxidation peak of [Fe(CN)_6_]^3−^ and reduction peak of [Fe(CN)_6_]^4−^. It was clear that the current signal of the NPG/GCE electrode was significantly higher than that of GCE. The above results indicated that NPG could promote electron transfer and improve the response current signal, which resulted in higher electron transfer efficiency and better electrochemical catalytic performance of the NPG/GCE electrode compared with bare GCE.

### 3.2. Sensitivity Detection of Uric Acid by the NPG/GCE

#### 3.2.1. The Detection Principle of Uric Acid by the NPG/GCE

**Figure 2:**
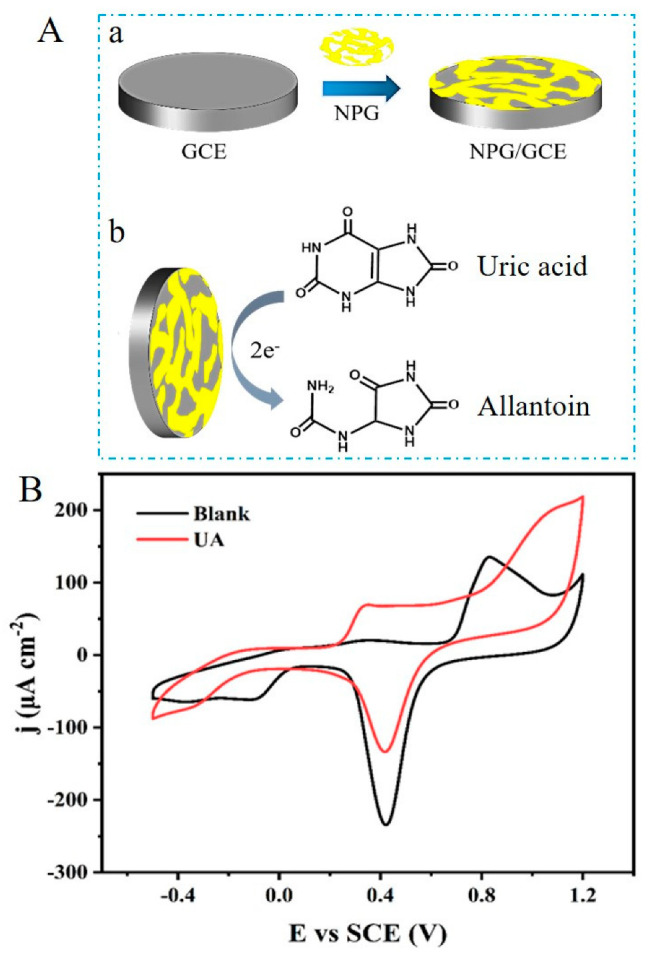
(**A**) Schematic diagram for the construction of NPG/GCE (a) and catalytic oxidation of uric acid by the NPG/GCE (b). (**B**) The CV curves of NPG/GCE in PBS (50 mM, pH 7.0) with and without 1.0 mM uric acid a scan rate of 50 mV s^−1^.

As shown in Figure 2A, uric acid is electrochemically oxidized by NPG to produce allantoin according to the following reaction mechanism. Uric acid undergoes a two-electron oxidation reaction in the dissociated state to form an unstable quinone anionic compound. When the pH of the electrolyte solution is ≥6, the ionic compound further undergoes a final decomposition reaction after two nucleophilic reactions with water molecules to produce the end-product allantoin [45]. The electrochemical oxidation process of uric acid is accompanied by the transfer of electrons, and the electrons generated in this process can be captured by the working electrode to form a current signal. Therefore, as the concentration of uric acid increases, the current signal captured by the NPG/GCE electrode will increase proportionally, achieving the quantitative detection of uric acid.

To verify the feasibility of NPG for detecting uric acid, the NPG/GCE was characterized by cyclic voltammetry in PBS (50 mM, pH 7.0) with and without 1.0 mM uric acid. Figure 2B showed a significant oxidation peak of uric acid around +0.4 V for the NPG/GCE electrode, which indicated the efficient electrochemical oxidation of uric acid on the surface of the NPG/GCE electrode generating a significant peak current response. In addition, the reduction peak of NPG was significantly reduced, which may be attributed to the fact that the transition from the oxidization state to the reduction state of gold atoms will lead to the decrease in the reduction peak for NPG during the electrochemical oxidation process of uric acid. These results demonstrated that the prepared NPG/GCE electrode exhibited good electrochemical oxidation activity for uric acid and can be used for the electrochemical detection of uric acid.

#### 3.2.2. The Electrochemical Detection of Uric Acid by the NPG/GCE

**Figure 3:**
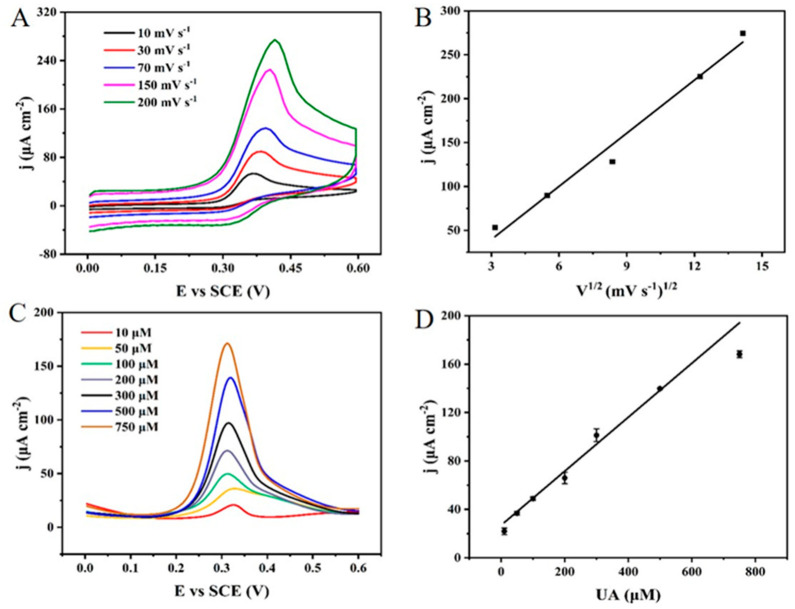
(**A**) The CV curves of the NPG/GCE in PBS (50 mM, pH 6.0) containing 1.0 mM uric acid at different scan rates. (**B**) The linear relationship between the oxidation peak current density of uric acid and the square root of scan rate for the electrooxidation of 1.0 mM uric acid. (**C**) The DPV curves of the NPG/GCE in PBS (50 mM, pH 6.0) containing different concentrations (10 to 750 μM) of uric acid. (**D**) The linear relationship between the oxidation peak current density of uric acid and uric acid concentration.

Based on the above results, the electrochemical oxidation behavior of uric acid at the surface of NPG/GCE was investigated by CV at different scan rates ranging from 10 mV s^−1^ to 200 mV s^−1^ in PBS (50 mM, pH 6.0) containing 1.0 mM uric acid. As shown in Figure 3A, the oxidation peak current density of uric acid gradually increased with the increase in the scan rate. According to the fitted curve in Figure 3B, a linear relationship was observed between the oxidation peak current density of uric acid and the square root of the scan rate, and the linear regression equation was: j (μA cm^−2^) = 20.17465 × V^1/2^ (mV^1/2^ s^−1/2^) − 20.94554, (R^2^ = 0.977). This result demonstrated that the electrochemical oxidation reaction of uric acid catalyzed by NPG was a diffusion-controlled type, which was consistent with the previous studies reported by Reddy et al. [46] and Abrori et al. [47].

The electrochemical detection of uric acid was carried out using differential pulse voltammetry (DPV) by the NPG/GCE in PBS (50 mM, pH 6.0) containing 10~750 μM uric acid. As shown in Figure 3C, the peak current density of uric acid increased with the increase in uric acid concentration. Figure 3D depicted the linear fitting curve between the peak current density and uric acid concentration, which suggested a good linear relationship in the range from 10 μM to 750 μM. The linear equation was j (μA cm^−2^) = 0.22291 × C_UA_ (μM) + 26.94179 (R^2^ = 0.986), with a high sensitivity of 222.91 μA mM^−1^ cm^−2^ and a LOD of 0.06 μM, which indicated that the NPG/GCE electrode constructed in this study had a good sensing performance in the detection of uric acid. Additionally, the NPG/GCE electrode has a wider detection range and lower LOD than most of the reported non-enzymatic electrochemical sensors for uric acid detection as shown in Table S1 (Supplementary Materials). Although the lower limit of the linear dynamic range for the NPG/GCE electrode is not as low as the reported electrochemical sensors in Table S1 such as pCu_2_O/rGO/GCE [48], Poly(DA)/PPY/GCE [31], Poly (β-CD)/CQDs/GCE [49], ZnO/rGO/CGE [50], and ZnO/rGO/SPE [51], the wider linear dynamic range makes it easier to meet the requirements for the practical application in uric acid detection (240 to 520 μM in serum and 1.44 to 4.43 mM in urine). Compared to the complexity of the electrode materials reported in Table S1 (Supplementary Materials), the preparation of NPG and NPG/GCE electrode are simpler, which is more promising for practical applications and large-scale production.

### 3.3. Sensitivity Detection of Creatinine by the NPG/GCE

#### 3.3.1. The Detection Principle of Creatinine by the NPG/GCE

**Figure 4:**
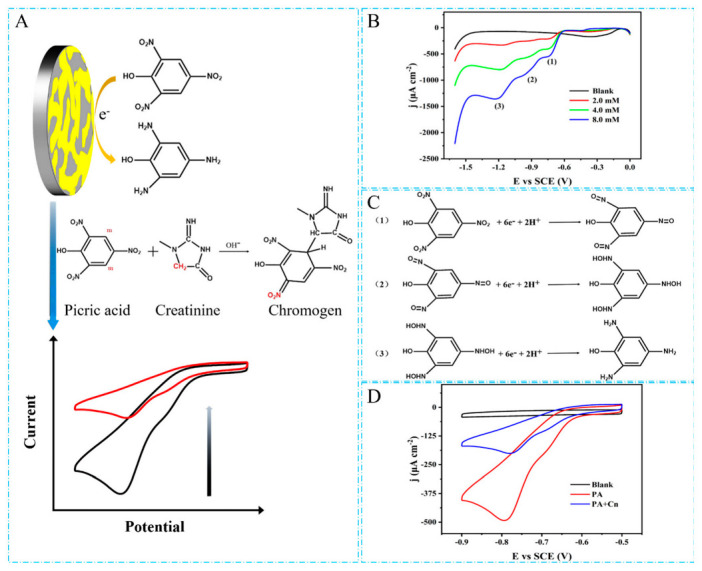
(**A**) Catalytic reduction of picric acid by the NPG/GCE and Jaffé reaction between picric acid and creatinine under alkaline conditions. (**B**) The CV curves of the NPG/GCE in NaOH (0.2 M) containing picric acid (2.0 mM, 4.0 mM, and 8.0 mM) at a scan rate of 50 mV s^−1^. (**C**) The specific reaction mechanism of picric acid catalyzed by NPG. (**D**) The CV response of picric acid before and after adding creatinine.

The detection principle of creatinine by the NPG/GCE electrode was demonstrated in Figure 4A. The NPG could not catalyze creatinine but showed good catalytic activity for picric acid. Since creatinine and picric acid could produce the Jaffé reaction under alkaline conditions [52], the amount of picric acid consumed by the Jaffé reaction would increase with the increase in creatinine concentration. Thus, the detection of creatinine could be indirectly achieved by determining the amount of picric acid consumed in the Jaffé reaction (Figure 4A).

NPG/GCE was characterized in NaOH (0.2 M) solutions containing different concentrations (2.0 mM, 4.0 mM, 8.0 mM) of picric acid using the CV method. As shown in Figure 4B, As shown in Figure 4B, three obvious reduction peaks of picric acid around −0.8 V, −0.9 V, and −1.2 V were observed, respectively, which indicated the reduction reaction of picric acid on the surface of the NPG/GCE electrode occurred with electron transfer, generating obvious peak current signals. Moreover, the catalytic reaction of picric acid by NPG was a complex multi-step process, which involved three consecutive electron transfer processes in the reduction process according to a previous report [53]. The reaction mechanism is shown in Figure 4C. Firstly, three nitro groups in the picric acid molecule were reduced to nitroso with the participation of two electrons and two protons, respectively, as described in Equation (1). Then, the nitroso was further reduced to the hydroxylamine group with the participation of electrons and protons as shown in Equation (2). Finally, the hydroxylamine group continued to be reduced and produced an amino group (Equation (3)). Figure 4B showed that the peak current signals corresponding to the three reduction peaks increased with the increase in picric acid concentration, which indicated that the NPG/GCE had good catalytic activity for picric acid. To protect the electrode and reduce the CV detection time, the first reduction process of picric acid at −0.8 V was used in subsequent experiments to indirectly detect creatinine in a potential window from −0.9 V to −0.5 V.

Figure 4D showed the CV curves of the electrical signal change in picric acid before and after the addition of creatinine detected by the NPG/GCE. Compared with the blank of 0.2 M NaOH, a significant reduction peak appeared at −0.8 V after the addition of 4.0 mM picric acid. Then 2.0 mM creatinine was added, and the measurement was performed after 20 min of reaction, and the current signal value of the picric acid reduction peak decreased significantly. These indicated that the NPG/GCE responded sensitively to the change in picric acid content in the Jaffé reaction, and that the constructed NPG/GCE sensor could be used to achieve reliable detection of creatinine.

#### 3.3.2. The Electrochemical Detection of Creatinine by the NPG/GCE

**Figure 5:**
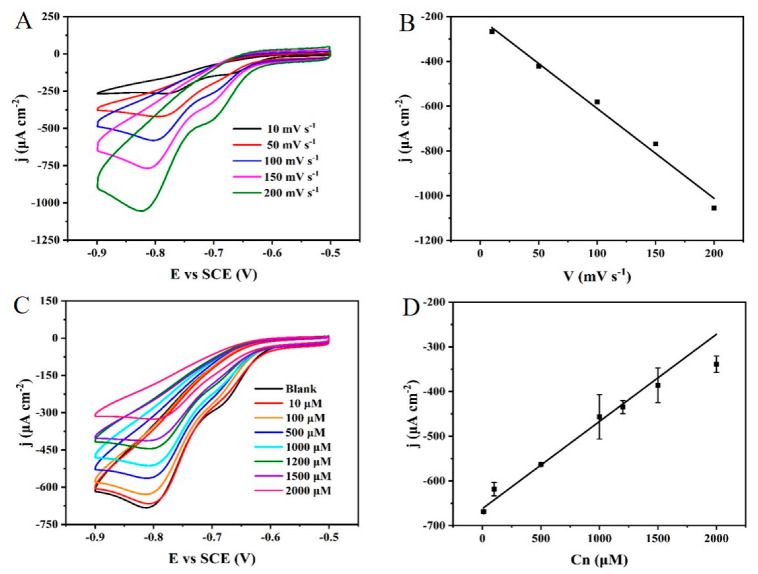
(**A**) The CV curves of the NPG/GCE in NaOH (0.2 M) containing 4.0 mM picric acid at different scan rates. (**B**) The linear relationship between the reduction peak current density of picric acid and the scan rate. (**C**) The CV curves of the NPG/GCE in NaOH (0.2 M) containing 4 mM picric acid and different concentrations of creatinine (**D**) The linear relationship between the reduction peak current density of picric acid and creatinine concentration.

To investigate the electrochemical behavior of picric acid reduction at the surface of the NPG/GCE, the CV curves of the NPG/GCE were recorded by varying the scan rate from 10 to 200 mV s^−1^ in NaOH (0.2 M) containing 4.0 mM picric acid. As shown in Figure 5A, the reduction peak current density of picric acid at −0.8 V increased with the increasing scan rate. Figure 5B described that the reduction peak current density of picric acid was linearly related to the scan rate with a linear equation: j (μA cm^−2^) = − 4.01353 × V (mV s^−1^) − 209.12167. These results indicated that the catalytic reaction of picric acid by the NPG/GCE electrode was a surface-controlled type.

The indirect electrochemical detection of creatinine was explored in NaOH solution (0.2 M) containing 4.0 mM picric acid using the CV method at a scan rate of 50 mV s^−1^. As shown in Figure 5C, the reduction peak current of picric acid detected by the NPG/GCE gradually decreased with the increase in creatinine concentration. Figure 5D demonstrated a good linear relationship between the creatinine concentration and the reduced peak current density, with a linear fitting equation: j (μA cm^−2^) = 0.19505 × C_Cn_ (μM) − 662.07238, R^2^ = 0.974 as well as a sensitivity of 195.05 μA mM^−1^ cm^−2^ and a LOD of 10 μM. In practical applications, the creatinine levels in the serum (45 to 140 μM) and urine (0.8 to 2.0 g/24 h) vary widely and fluctuate significantly, which requires a wide detection range, a low detection line, and a high sensitivity for electrochemical sensors. Compared with the reported electrochemical sensors for creatinine detection as shown in Table S2 (Supplementary Materials), such as GCE [32] (indirect detection based on electrochemical catalysis of picric acid), electrodeposition Cu/SPCE [54], Fc-enzy.ink/SPCE [55], and CuNPs/PDA-rGO-NB/GCE [56], the NPG/GCE electrode exhibited a wider detection range with a lower LOD, which makes it more suitable for practical detection requirements. Additionally, an improved linear dynamic range has been achieved by the electrochemical sensor based on ZIF-8 NPs/PEDOT:PSS/ITO [57]. Compared with the electrochemical sensor constructed by ZIF-8 NPs/PEDOT:PSS/ITO [57], the NPG/GCE electrode has a comparable linear dynamic range with a lower LOD. Especially, the simple fabrication and cost efficiency of the NPG/GCE electrode may make it more competitive in practical applications.

### 3.4. Anti-Interference and Application in Real Sample Detection of the NPG/GCE

#### 3.4.1. Anti-Interference of Uric Acid Detection

**Figure 6:**
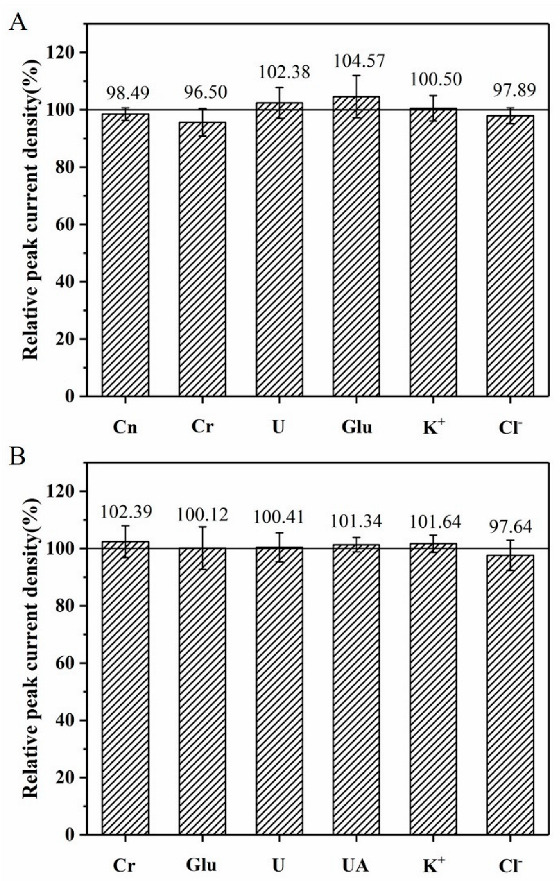
Influence of common compounds and ions in urine on determination of uric acid (**A**) and creatinine (**B**).

As one of the most important parameters to evaluate the detection performance of electrochemical sensors, the anti-interference ability of the NPG/GCE was investigated by recording the electrochemical response of uric acid in the presence of the common interferences in urine such as creatinine, creatine, urea, glucose, K^+^, and Cl^−^. Anti-interference studies were performed by adding 1.5 mM interferents individually into PBS (50 mM, pH 6.0) containing 300 μM uric acid. The relative peak current density values were calculated after the addition of different interferents, respectively. As shown in Figure 6A, there were no significant effects on the electrochemical response of uric acid catalyzed by the NPG/GCE electrode after the addition of interferents. The relative peak current density values ranged from 96.50% to 104.57%, indicating that these interferents did not affect the detection of uric acid by the NPG/GCE electrode and the NPG/GCE electrode exhibited good anti-interference capability.

#### 3.4.2. Anti-Interference of Creatinine Detection

To investigate the effect of some common compounds and ions in human urine on the detection of creatinine by the NPG/GCE electrode, 1.5 mM of common interferents (creatine, glucose, K^+^, Cl^−^, urea, and uric acid) were added to NaOH (0.2 M) containing 300 μM creatinine and 4.0 mM picric acid, respectively. The CV method was used to record the peak current density values before and after the addition of interferents and the relative peak current density values were calculated accordingly. As shown in Figure 6B, the reduction peak current density of picric acid did not change significantly when these interfering substances were present in the reaction solution, and the relative peak current density values were between 97.64% and 102.39%. These results demonstrated that these interferents examined had no significant effect on the indirect detection of creatinine by the NPG/GCE electrode under the established conditions.

#### 3.4.3. The Detection of Uric Acid in Human Urine

In order to further evaluate the application potential of the NPG/GCE electrode in real samples, human urine samples were analyzed by the NPG/GCE electrode and HPLC, respectively. As shown in Table 1, the concentrations of uric acid in human urine samples detected by the NPG/GCE electrode were consistent with the results obtained by HPLC, and the deviations were less than 10.28%. To further evaluate the accuracy of uric acid detection by the NPG/GCE electrode, different concentrations of uric acid were added to the three human urine samples, and then detected by the NPG/GCE electrode. The recoveries of the spiked uric acid ranged from 89% to 118% (Table 1). These results indicated that the NPG/GCE sensor had good accuracy and reproducibility for the detection of uric acid, exhibiting great potential for urine analysis.

#### 3.4.4. The Detection of Creatinine in Human Urine

The potential of creatinine detection in human urine samples by the NPG/GCE electrode was also analyzed by the NPG/GCE electrode and HPLC, respectively. Compared with the results of HPLC (Table 2), the deviation rates of creatinine in human urine detected by the NPG/GCE electrode were less than 4.17%. To further evaluate the accuracy of creatinine detection by the NPG/GCE electrode, different concentrations of creatinine were added to the three human urine samples and then detected by the NPG/GCE electrode. The recoveries of the spiked creatinine were between 92.50% and 117.40%, suggesting the reliability and repeatability of creatinine detection by the NPG/GCE electrode.

## 4. Conclusions

In conclusion, based on the unique electrochemical catalytic activity of NPG for uric acid and picric acid, a non-enzyme multifunctional electrochemical sensor (the NPG/GCE) was successfully constructed for the direct detection of uric acid and indirect detection of creatinine. The proposed NPG/GCE electrochemical sensor exhibited good analytical properties both in uric acid and creatinine detections. The sensitive analysis of uric acid and creatinine in human urine were achieved using the NPG/GCE electrochemical sensor with an accuracy comparable to that of HPLC. Compared with the existing electrochemical sensors for uric acid and creatinine detection, the NPG/GCE electrochemical sensor exhibited wider linear dynamic ranges and lower LOD, which could meet the detection requirements of uric acid and creatinine in practical applications. Importantly, the wider linear dynamic ranges of the NPG/GCE electrochemical sensor alleviate the problem of low detection accuracy in uric acid and creatinine analysis caused by sample dilution due to the narrow linear ranges of existing electrochemical detection sensors. In addition, the proposed NPG/GCE electrochemical sensor was constructed only by NPG. The excellent structural and functional properties of NPG endowed the proposed electrochemical sensor with many advantages, including high stability, real sample detection applicability, simple fabrication, cost efficiency, and flexible adaptability, which makes it a potential candidate for urine analysis.

## Figures and Tables

**Table 1:** The detection of uric acid in human urine samples.

Urine Samples	Detected by NPG/GCE (mM)	Detected by HPLC (mM)	Deviation Rate (%)	Spiked of Uric Acid (mM)	Detected by NPG/GCE (mM)	Recovery Rate (%)
1	1.17 ± 0.02	1.11 ± 0.03	5.40	1.0	2.24 ± 0.16	107.00
				2.0	3.26 ± 0.03	104.50
				5.0	5.93 ± 0.10	95.20
2	1.92 ± 0.04	2.14 ± 0.08	−10.28	1.0	3.04 ± 0.20	112.00
				2.0	4.28 ± 0.01	118.00
				5.0	6.92 ± 0.22	100.00
3	0.95 ± 0.01	0.90 ± 0.01	5.56	1.0	2.03 ± 0.04	108.00
				2.0	2.89 ± 0.12	97.00
				5.0	5.40 ± 0.28	89.00

**Table 2:** The detection of creatinine in human urine samples.

Urine Samples	Detected by NPG/GCE (mM)	Detected by HPLC (mM)	Deviation Rate (%)	Spiked of Creatinine (mM)	Detected by NPG/GCE (mM)	Recovery Rate (%)
1	7.65 ± 0.61	7.42 ± 0.12	3.09	2.0	9.78 ± 0.23	106.50
				5.0	12.77 ± 0.96	102.40
				10.0	17.71 ± 1.65	100.60
2	5.58 ± 0.08	5.66 ± 0.11	−1.41	2.0	7.75 ± 0.41	108.50
				5.0	11.45 ± 0.77	117.40
				10.0	14.96 ± 0.90	93.80
3	4.60 ± 0.74	4.80 ± 0.04	−4.17	2.0	6.45 ± 0.49	92.50
				5.0	10.02 ± 0.82	108.40
				10.0	15.00 ± 0.49	104.00

## Data Availability

Data is contained within the article or Supplementary Materials.

## Supplementary Materials

The following supporting information can be downloaded at: https://www.mdpi.com/article/10.3390/bios12080588/s1. Table S1. Performance comparison of uric acid detection sensors. Table S2. Performance comparison of creatinine detection sensors.

## References

[1] Yang Y.D. (1998). Simultaneous determination of creatine, uric acid, creatinine and hippuric acid in urine by high performance liquid chromatography. Biomed. Chromatogr..

[2] Hernandez-Ramirez D., Mendoza-Huizar L.H., Galan-Vidal C.A., Aguilar-Lira G.Y., Alvarez-Romero G.A. (2022). Review-trends in the development of non-enzymatic electrochemical sensors modified with a metal-organic framework for quantification of uric acid. J. Electrochem. Soc..

[3] Roopa R.A., Mantelingu K., Guin M., Thimmaiah S.B. (2022). Bienzymatic spectrophotometric method for uric acid estimation in human serum and urine. J. Anal. Chem..

[4] Liu J., Xu C., Ying L., Zang S., Zhuang Z., Lv H., Yang W., Luo Y., Ma X., Wang L. (2017). Relationship of serum uric acid level with non-alcoholic fatty liver disease and its inflammation progression in non-obese adults. Hepatol. Res..

[5] Perticone M., Tripepi G., Maio R., Cimellaro A., Addesi D., Baggetta R., Sciacqua A., Sesti G., Perticone F. (2017). Risk reclassification ability of uric acid for cardiovascular outcomes in essential hypertension. Int. J. Cardiol..

[6] Prasad M., Matteson E.L., Herrmann J., Gulati R., Rihal C.S., Lerman L.O., Lerman A. (2017). Uric acid is associated with inflammation, coronary microvascular dysfunction, and adverse outcomes in postmenopausal women. Hypertension.

[7] Pellecchia M.T., Savastano R., Moccia M., Picillo M., Siano P., Erro R., Vallelunga A., Amboni M., Vitale C., Santangelo G. (2016). Lower serum uric acid is associated with mild cognitive impairment in early Parkinson’s disease: A 4-year follow-up study. J. Neural Transm..

[8] Kubota Y., McAdams-DeMarco M., Folsom A.R. (2016). Serum uric acid, gout, and venous thromboembolism: The atherosclerosis risk in communities study. Thromb. Res..

[9] Kanbay M., Jensen T., Solak Y., Le M., Roncal-Jimenez C., Rivard C., Lanaspa M.A., Nakagawa T., Johnson R.J. (2016). Uric acid in metabolic syndrome: From an innocent bystander to a central player. Eur. J. Intern. Med..

[10] Tsikas D., Wolf A., Mitschke A., Gutzki F.M., Will W., Bader M. (2010). GC-MS determination of creatinine in human biological fluids as pentafluorobenzyl derivative in clinical studies and biomonitoring: Inter-laboratory comparison in urine with Jaffe, HPLC and enzymatic assays. J. Chromatogr. B.

[11] Liotta E., Gottardo R., Bonizzato L., Pascali J.P., Bertaso A., Tagliaro F. (2009). Rapid and direct determination of creatinine in urine using capillary zone electrophoresis. Clin. Chim. Acta.

[12] Niesser M., Koletzko B., Peissner W. (2012). Determination of creatinine in human Urine with flow injection tandem mass spectrometry. Ann. Nutr. Metab..

[13] Kumar P., Jaiwal R., Pundir C.S. (2017). An improved amperometric creatinine biosensor based on nanoparticles of creatininase, creatinase and sarcosine oxidase. Anal. Biochem..

[14] Sarkar K. (2021). A Review on the development of spectroscopic sensors for the detection of creatinine in human blood serum. Int. J. Life Sci. Pharma Res..

[15] Pundir C.S., Kumar P., Jaiwal R. (2019). Biosensing methods for determination of creatinine: A review. Biosens. Bioelectron..

[16] Talalak K., Noiphung J., Songjaroen T., Chailapakul O., Laiwattanapaisal W. (2015). A facile low-cost enzymatic paper-based assay for the determination of urine creatinine. Talanta.

[17] Gamagedara S., Shi H., Ma Y. (2012). Quantitative determination of taurine and related biomarkers in urine by liquid chromatography-tandem mass spectrometry. Anal. Bioanal. Chem..

[18] Magalhaes J., Machado A. (2002). Array of potentiometric sensors for the analysis of creatinine in urine samples. Analyst.

[19] Income K., Ratnarathorn N., Khamchaiyo N., Srisuvo C., Ruckthong L., Dungchai W. (2019). Disposable nonenzymatic uric acid and creatinine sensors using mu PAD coupled with screen-printed reduced graphene oxide-gold nanocomposites. Int. J. Anal. Chem..

[20] Jin D., Seo M.-H., Bui The H., Quoc-Thai P., Conte M.L., Thangadurai D., Lee Y.-I. (2016). Quantitative determination of uric acid using CdTe nanoparticles as fluorescence probes. Biosens. Bioelectron..

[21] Pezzaniti J.L., Jeng T.W., McDowell L., Oosta G.M. (2001). Preliminary investigation of near-infrared spectroscopic measurements of urea, creatinine, glucose, protein, and ketone in urine. Clin. Biochem..

[22] Zinellu A., Sotgia S., Zinellu E., Chessa R., Deiana L., Carru C. (2006). Assay for the simultaneous determination of guanidinoacetic acid, creatinine and creatine in plasma and urine by capillary electrophoresis UV-detection. J. Sep. Sci..

[23] Zuo Y., Wang C., Zhou J., Sachdeva A., Ruelos V.C. (2008). Simultaneous determination of creatinine and uric acid in human urine by High-Performance Liquid Chromatography. Anal. Sci..

[24] Ma X., Ran X., Meng L., Li L., Mao X. (2014). Simultaneous determination of creatine and uric acid in urine and serum samples by reversed-phase high performance liquid chromatographic method. Chin. J. Anal. Lab..

[25] Xing X., Shi X., Zhang M., Jin W., Ye J. (2008). CE determination of creatinine and uric acid in saliva and urine during exercise. Chromatographia.

[26] See H.H., Schmidt-Marzinkowski J., Pormsila W., Morand R., Kraehenbuehl S., Hauser P.C. (2012). Determination of creatine and phosphocreatine in muscle biopsy samples by capillary electrophoresis with contactless conductivity detection. Anal. Chim. Acta.

[27] Ma Q.S., Wang Q., Zhao K.S., Zhai S.B., Liu S., Liu Z.Q. (2013). UPLC-MS/MS method for determination of uric acid and creatinine in serum and urine of hyperuricemic mice. Chem. J. Chin. Univ.-Chin..

[28] Wang J.-M., Chu Y., Li W., Wang X.-Y., Guo J.-H., Yan L.-L., Ma X.-H., Ma Y.-L., Yin Q.-H., Liu C.-X. (2014). Simultaneous determination of creatine phosphate, creatine and 12 nucleotides in rat heart by LC-MS/MS. J. Chromatogr. B.

[29] Tang J., Hui Z.Z., Hu T., Cheng X., Guo J.H., Li Z.R., Yu H. (2022). A sensitive acetaminophen sensor based on Co metal-organic framework (ZIF-67) and macroporous carbon composite. Rare Met..

[30] Hu J.-Y., Li Z., Zhai C.-Y., Wang J.-F., Zeng L.-X., Zhu M.-S. (2021). Plasmonic photo-assisted electrochemical sensor for detection of trace lead ions based on Au anchored on two-dimensional g-C3N4/graphene nanosheets. Rare Met..

[31] Adeosun W.A., Asiri A.M., Marwani H.M., Rahman M.M. (2020). Enzymeless electrocatalytic detection of uric acid using polydopamine/polypyrrole copolymeric film. ChemistrySelect.

[32] de Araujo W.R., Salles M.O., Paixao T.R.L.C. (2012). Development of an enzymeless electroanalytical method for the indirect detection of creatinine in urine samples. Sens. Actuator B-Chem..

[33] Stefan R.I., Bokretsion R.G. (2003). Determination of creatine and creatinine using a diamond paste based electrode. Instrum. Sci. Technol..

[34] Jayasekhar Babu P., Tirkey A., Mohan Rao T.J., Chanu N.B., Lalchhandama K., Singh Y.D. (2022). Conventional and nanotechnology based sensors for creatinine (A kidney biomarker) detection: A consolidated review. Anal. Biochem..

[35] Qiu H.J., Li X., Xu H.T., Zhang H.J., Wang Y. (2014). Nanoporous metal as a platform for electrochemical and optical sensing. J. Mater. Chem. C.

[36] Li X., Xing Y., Xu Y., Deng Q., Zhang K., Shao L.-H. (2019). Hierarchical nanoporous carbon templated and catalyzed by the bicontinuous nanoporous copper for high performance electrochemical capacitors. ChemistrySelect.

[37] Cao Z., Zhou T., Ma X., Shen Y., Deng Q., Zhang W., Zhao Y. (2020). Hydrogen production from urea sewage on NiFe-based porous electrocatalysts. ACS Sustain. Chem. Eng..

[38] Han Z., Qi Z., Wei Q., Deng Q., Wang K. (2020). The mechanical effect of MnO_2_ layers on electrochemical actuation performance of nanoporous gold. Nanomaterials.

[39] Ding Y., Chen M.W. (2009). Nanoporous metals for catalytic and optical applications. MRS Bull..

[40] Wang K., Ding Y. (2020). Carbon-free nanoporous gold based membrane electrocatalysts for fuel cells. Prog. Nat. Sci..

[41] Gao Y., Ding Y. (2020). Nanoporous metals for heterogeneous catalysis: Following the success of raney nickel. Chem.-Eur. J..

[42] Zhang X., Ding Y. (2013). Unsupported nanoporous gold for heterogeneous catalysis. Catal. Sci. Technol..

[43] Xiao S., Shang K.S., Li W.J., Wang X. (2022). An efficient biosensor based on the synergistic catalysis of Helicobacter pylori urease b subunit and nanoplatinum for urease inhibitors screening and antagonistic mechanism analyzing. Sens. Actuator B-Chem..

[44] Zhang Y., Gao Y., Zhang X., Wang H., Xia T., Bian C., Liang S., Tang X., Wang X. (2019). Electrochemical immunosensor for HBe antigen detection based on a signal amplification strategy: The co-catalysis of horseradish peroxidase and nanoporous gold. Sens. Actuators B-Chem..

[45] Lakshmi D., Whitcombe M.J., Davis F., Sharma P.S., Prasad B.B. (2011). Electrochemical dtection of uric acid in mixed and clinical samples: A Review. Electroanalysis.

[46] Reddy Y.V.M., Sravani B., Agarwal S., Gupta V.K., Madhavi G. (2018). Electrochemical sensor for detection of uric acid in the presence of ascorbic acid and dopamine using the poly(DPA)/SiO_2_@Fe_3_O_4_ modified carbon paste electrode. J. Electroanal. Chem..

[47] Abrori S.A., Septiani N.L.W., Hakim F.N., Maulana A., Suyatman, Nugraha, Anshori I., Yuliarto B. (2021). Non-enzymatic electrochemical detection for uric acid based on a glassy carbon electrode modified with MOF-71. IEEE Sens. J..

[48] Mei L.P., Feng J.J., Wu L., Chen J.R., Shen L., Xie Y., Wang A.J. (2016). A glassy carbon electrode modified with porous Cu_2_O nanospheres on reduced graphene oxide support for simultaneous sensing of uric acid and dopamine with high selectivity over ascorbic acid. Microchim. Acta.

[49] Chen J., He P., Bai H., He S., Zhang T., Zhang X., Dong F. (2017). Poly(beta-cyclodextrin)/carbon quantum dots modified glassy carbon electrode: Preparation, characterization and simultaneous electrochemical determination of dopamine, uric acid and tryptophan. Sens. Actuator B-Chem..

[50] Zhang X., Zhang Y.C., Ma L.X. (2016). One-pot facile fabrication of graphene-zinc oxide composite and its enhanced sensitivity for simultaneous electrochemical detection of ascorbic acid, dopamine and uric acid. Sens. Actuator B-Chem..

[51] Rezaei R., Foroughi M.M., Beitollahi H., Alizadeh R. (2018). Electrochemical sensing of uric acid using a ZnO/graphene nanocomposite modified graphite screen Printed electrode. Russ. J. Electrochem..

[52] Hussain I., Tariq M.I., Siddiqui H.L. (2009). Structure elucidation of chromogen resulting from Jaffe’s reaction. J. Chem. Soc. Pak..

[53] Wang Y., Cao W., Wang L., Zhuang Q., Ni Y. (2018). Electrochemical determination of 2,4,6-trinitrophenol using a hybrid film composed of a copper-based metal organic framework and electroreduced graphene oxide. Microchim. Acta.

[54] Raveendran J., Resmi P.E., Ramachandran T., Nair B.G., Babu T.G.S. (2017). Fabrication of a disposable non-enzymatic electrochemical creatinine sensor. Sens. Actuator B-Chem..

[55] Chen P., Peng Y., He M., Yan X.-C., Zhang Y., Liu Y.-N. (2013). Sensitive electrochemical detection of creatinine at disposable screen-printed carbon electrode mixed with ferrocenemethanol. Int. J. Electrochem. Sci..

[56] Gao X.H., Gui R.J., Guo H.J., Wang Z.H., Liu Q.Y. (2019). Creatinine-induced specific signal responses and enzymeless ratiometric electrochemical detection based on copper nanoparticles electrodeposited on reduced graphene oxide-based hybrids. Sens. Actuator B-Chem..

[57] Chakraborty T., Das M., Lin C.Y., Su Y., Yuan B., Kao C.H. (2022). ZIF-8 Nanoparticles based electrochemical sensor for non-enzymatic creatinine detection. Membranes.

