# Supplementary material for: Sensitivity Detection of Uric Acid and Creatinine in Human Urine Based on Nanoporous Gold
Source: Biosensors (Basel). 2022 Aug 1;12(8):588. doi: 10.3390/bios12080588 (PMC9405689; doi:10.3390/bios12080588)
Supplement: Supplementary file 1 [file biosensors-12-00588-s001.zip › biosensors-1803826-supplementary.pdf]

Supplementary Material

Sensitivity Detection of Uric Acid and Creatinine in Human Urine Based on Nanoporous Gold

Keshuai Shang [1], Shuangjue Wang [1], Siyu Chen [2] and Xia Wang [1,*]

[1] State Key Laboratory of Microbial Technology, Shandong University, Qingdao 266237, China; asakas13468242423@163.com (K.S.); wsj782872917@163.com (S.W.)

[2] The Faculty of Engineering, Architecture and Information Technology, The University of Queensland, Brisbane, QLD 4072, Australia; siyu.chen2@uqconnect.edu.au

* Correspondence: ghwx@sdu.edu.cn; Tel: +86-130-7534-3592

Table S1. Performance comparison of uric acid detection sensors.

Electrode materials	Sensor type	Detection method	Linear dynamic range （μM）	LOD （μM）	Refs.
pCu_2O/rGO/GCE	Non-Enzymatic	DPV	1.0~138	0.112	[48]
Poly(DA)/PPY/GCE	Non-Enzymatic	DPV	0.5~40	0.110	[31]
Poly (β-CD)/CQDs/GCE	Non-Enzymatic	DPV	0.3~200	0.010	[49]
ZnO/rGO/CGE	Non-Enzymatic	DPV	1~70	0.330	[50]
ZnO/rGO/SPE	Non-Enzymatic	DPV	1~100	0.430	[51]
NPG/GCE	Non-Enzymatic	DPV	10~750	0.060	This work

Table S2. Performance comparison of creatinine detection sensors

Electrode materials	Sensor type	Detection method	Linear dynamic range （μM）	LOD （μM）	Refs.
GCE	Non-Enzymatic	DPV	1~80	0.380	[32]
Electrodeposition Cu/SPCE	Non-Enzymatic	CV	6~378	0.074	[54]
Fc-enzy.ink/SPCE	Enzymatic	DPV	5~1000	2.400	[55]
CuNPs/PDA-rGO-NB/GCE	Non-Enzymatic	SWV	0.01~100	0.002	[56]
ZIF-8 NPs/PEDOT:PSS/ITO	Non-Enzymatic	EIS	50~2500	30.000	[57]
NPG/GCE	Non-Enzymatic	CV	10~2000	0.060	This work

References

31. Adeosun, W.A.; Asiri, A.M.; Marwani, H.M.; Rahman, M.M. Enzymeless electrocatalytic detection of uric acid using polydopamine/polypyrrole copolymeric film. *ChemistrySelect* **2020**, *5*, 156–164.
32. de Araujo, W.R.; Salles, M.O.; Paixao, T.R.L.C. Development of an enzymeless electroanalytical method for the indirect detection of creatinine in urine samples. *Sens. Actuator B-Chem.* **2012**, *173*, 847–851.
48. Mei, L.P.; Feng, J.J.; Wu, L.; Chen, J.R.; Shen, L.; Xie, Y.; Wang, A.J. A glassy carbon electrode modified with porous Cu_2O nanospheres on reduced graphene oxide support for simultaneous sensing of uric acid and dopamine with high selectivity over ascorbic acid. *Microchim. Acta* **2016**, *183*, 2039–2046.
49. Chen, J.; He, P.; Bai, H.; He, S.; Zhang, T.; Zhang, X.; Dong, F. Poly(beta-cyclodextrin)/carbon quantum dots modified glassy carbon electrode: Preparation, characterization and simultaneous electrochemical determination of dopamine, uric acid and tryptophan. *Sens. Actuator B-Chem.* **2017**, *252*, 9–16.
50. Zhang, X.; Zhang, Y.C.; Ma, L.X. One-pot facile fabrication of graphene-zinc oxide composite and its enhanced sensitivity for simultaneous electrochemical detection of ascorbic acid, dopamine and uric acid. *Sens. Actuator B-Chem.* **2016**, *227*, 488–496.
51. Rezaei, R.; Foroughi, M.M.; Beitollahi, H.; Alizadeh, R. Electrochemical sensing of uric acid using a ZnO/graphene nanocomposite modified graphite screen Printed electrode. *Russ. J. Electrochem.* **2018**, *54*, 860–866.
54. Raveendran, J.; Resmi, P.E.; Ramachandran, T.; Nair, B.G.; Babu, T.G.S., Fabrication of a disposable non-enzymatic electrochemical creatinine sensor. *Sens. Actuator B-Chem.* **2017**, *243*, 589-595.
55. Chen, P.; Peng, Y.; He, M.; Yan, X.-C.; Zhang, Y.; Liu, Y.-N., Sensitive electrochemical detection of creatinine at disposable screen-printed carbon electrode mixed with ferrocenemethanol. *Int. J. Electrochem. Sci.* **2013**, *8* , 8931-8939.
56. Gao, X. H.; Gui, R. J.; Guo, H. J.; Wang, Z. H.; Liu, Q. Y., Creatinine-induced specific signal responses and enzymeless ratiometric electrochemical detection based on copper nanoparticles electrodeposited on reduced graphene oxide-based hybrids. *Sens. Actuator B-Chem* **2019**, *285*, 201-208.
57. Chakraborty, T.; Das, M.; Lin, C. Y.; Su, Y.; Yuan, B.; Kao, C. H., ZIF-8 Nanoparticles based electrochemical sensor for non-enzymatic creatinine detection. *Membranes* **2022**, *12* .159.
